# The Role of Antioxidant Foods and Nutraceuticals in Ageing

**DOI:** 10.3390/antiox13070839

**Published:** 2024-07-13

**Authors:** Marco Giorgio, Maria Pia Rigobello

**Affiliations:** Department of Biomedical Sciences, University of Padua, Via Ugo Bassi 58/B, 35131 Padova, Italy

## 1. Introduction

The free radical theory of aging proposed by Denham Harman in the 1950s identified the accumulation of oxidative damage over time as a cause of aging [1]. Since then, the relationship between oxidative stress and aging processes has become the subject of ample research and extensive fake news as well.

The molecules responsible for these processes are partial-reduced oxygen species that can damage cellular components due to their high reactivity. As a consequence, the imbalance between reactive oxygen species (ROS) and the body’s ability to aggravate their harmful effects leads to a progressive accumulation of dysfunction, i.e., aging traits. In turn, the antioxidant capacity of an organism also plays a role in aging, thus accelerating the onset of life-threatening diseases. In this view, restoring balance through the intake of exogenous antioxidants appeared to be the strategy to prevent organisms from aging [2]. Oxidative stress was defined by H. Sies as an imbalance between oxidants and antioxidants [3]. This concept became so popular that antioxidants were used in foods and cosmetics that claimed to be anti-aging.

Common endogenous antioxidant defenses in eukaryotes include enzymes such as superoxide dismutase, catalase, and glutathione peroxidase, as well as free radical scavenger compounds such as glutathione, ascorbic acid, tocopherol, and beta-carotene. Studies in *C. elegans*, *Drosophila*, and mice have shown that enhancing antioxidant capacity extend lifespan, while others have shown no significant effect [4,5]. Various environmental factors, including exercise, exposure to pollutants, and of course diet, can influence both the expression of endogenous ROS scavengers and directly supply antioxidants, as in the case of a variety of nutraceuticals derived from plants. However, whether dietary antioxidants can reduce the risk of chronic diseases remains unclear [6], as the guidelines on antioxidant health claims on food labels are wary [7].

This Special Issue presents novel evidence from different perspectives that supports the potential role of antioxidant supplementation in human health and aging, while also underlining the complexity of the phenomenon.

## 2. Overview of Published Articles

The work of Bassolino et al. [Contribution 1] provides a good introduction to the controversy of diet efficacy, showing that clinical studies on the impact of antioxidant-rich foods, particularly those derived from cereals and the Solanaceae family, are still not sufficient to definitively prove their benefits on human health. While laboratory and animal studies often show promising results regarding the antioxidant properties of these foods and their potential to mitigate oxidative stress and inflammation, translating these findings to human health outcomes remains complex. Several factors contribute to this insufficiency: study design variability, compound bioavailability and metabolism, long-term vs short-term outcomes, complex food matrices, and confounding factors.

Reductionist approaches offer some hints to solve complex processes as suggested by Tonolo and colleagues [Contribution 2]. By using in silico tools, they identified antioxidant bioactive peptides that have the ability to prevent the action of oxidant species and to protect the body from oxidative stress activating specific stress response pathways. Indeed, a molecular docking approach would be useful to predict the capacity of bioactive peptides to interact with specific molecular pathways, such as Nrf2/Keap1. Bioactive peptides are typically derived from proteins through enzymatic hydrolysis, fermentation, or other biotechnological processes and can be found in a variety of food sources, including milk, eggs, fish, and soy and other plant-based proteins. Research on antioxidant bioactive peptides continues to evolve, with ongoing studies exploring their potential in various health applications. Following this, advancements in peptide synthesis and extraction techniques are expected to enhance the availability and efficacy of these compounds. Moreover, understanding the bioavailability and metabolism of these peptides in the human body appears crucial for optimizing their use in health and nutrition.

In simple “wet” models such as cell cultures, while carom extract has been found to have promising neuroprotective functions and a convincing effect on different pathways involved in neurodegeneration, as shown by Sharma and colleagues [Contribution 3], further research is needed to fully understand its mechanisms and efficacy in vivo. One step further, using *Caenorhabditis* in addition to cultured cells, Kim and colleagues [Contribution 4] revealed that the nutraceutical butein, with its diverse range of biological activities, holds significant promise as a longevity agent. Notably, although not exactly common in diet, both carom extract, derived from the seeds of *Trachyspermum ammi*, and butein, a chalcone found in the flame of the forest, blackwood, and the lacquer tree, have been traditionally used in various medicinal systems for their health benefits. These two substances, as is often the case for plant derivatives, act in different chemical ways, such as chelation, antioxidation, and specific ligands; therefore, the claim of antioxidants lacks accuracy by nature.

Vitheejongjaroen and colleagues [Contribution 5] provide further evidence of the complexity of diet–phenotype interaction, suggesting that the probiotic *Bifidobacterium animalis* strain MSMC83, which remodels the microbiota, has potential health benefits, especially against inflammation, while improving antioxidant capacity in rats.

*Mangifera indica*, commonly known as the mango tree, is well known for its delicious fruit, but its leaves also possess significant microbiological activity and health benefits. Several studies have focused on the polyphenols content of mango leaves, revealing their potential effects on the gut microbiota, inhibition of pathogenic microbes, and antioxidant activity. Sferrazzo et al. [Contribution 6] investigated the subclass of benzophenone–xanthone metabolites present in mango leaves, revealing they exert antioxidant and antibacterial activities toward specific bacterial species that may be involved in reducing gut inflammation.

Particularly important for aging-associated diseases are the studies on neuroprotective phytochemicals. *Hericium erinaceus*, commonly known as lion’s mane mushroom, is a medicinal mushroom that has been traditionally used in various cultures for its health-promoting properties. Recent scientific research has highlighted its potential benefits in neuroprotection and the management of neurodegenerative diseases. One of the key mechanisms through which *Hericium erinaceus* exerts its neuroprotective effects is through the activation of the Nrf2 (nuclear factor erythroid 2-related factor 2) pathway as reported by Lee et al. [Contribution 7]. The antioxidant effects of Nrf2 activation by *Hericium erinaceus* lead to decreased oxidative damage and inflammation, contributing to the preservation of neuronal integrity and function. By enhancing antioxidant defenses, reducing inflammation, and promoting neuronal health and regeneration in a model of brain injury, *Hericium erinaceus* extract may play a role in the prevention and management of conditions such as Alzheimer’s and Parkinson’s diseases. Interestingly, the activation of the Nrf2 pathway is an emerging target of effective nutraceuticals as it enhances the production of superoxide dismutase, catalase, and heme oxygenase-1, which help mitigate oxidative stress. 

## 3. Conclusions

The relationship between antioxidants and longevity is complex. While epidemiological studies suggest that a diet rich in antioxidants from fruits and vegetables is associated with a reduced risk of chronic diseases and a potentially longer lifespan, clinical trials on antioxidant supplements have yielded mixed results. Some studies show benefits in reducing disease risk, while others indicate no significant impact on longevity or, in some cases, potential harm. So far, the benefits of antioxidant nutraceuticals are less clear and may depend on various factors, including dosage and the individual’s overall health status. 

At the biochemical level, ROS-mediated oxidation plays a dual role, potentially damaging cells but also acting as a signaling mechanism for beneficial adaptive responses. Therefore, excessive antioxidant supplementation might interfere with beneficial oxidative signaling and adaptive responses, abolishing the hormetic effect dependent on low amounts of ROS. The results discussed in the present Special Issue uncover the multiple sites of action of potential healthy nutraceuticals and support continued research for better understanding the optimal sources and amounts of antioxidants for slowing aging and its associated diseases. 
